# Reply

**DOI:** 10.1016/j.jvsv.2026.102547

**Published:** 2026-06-02

**Authors:** Luis Moreno, Fredy Rivero, Nicolas Forero Ramirez, Luis Felipe Cabrera Vargas

**Affiliations:** CEO and Vascular Surgeon, Clinica Vascular Moreno, Cúcuta, Colombia; Cirujano Vascular, Clínica Vascular Moreno, Cúcuta, Colombia; Médico General, Universidad de Los Andes, Bogotá, Colombia; Departamento de Cirugía, Sección de Cirugía Vascular, Fundación Santa Fe de Bogotá, Bogota, Colombia

We appreciate Professor Mark S. Whiteley's thoughtful comments on our article, “A Novel Endovascular Thermal Ablation Technique for Pelvic Venous Disorders via Basilic Vein Access: A Prospective Descriptive Study.”^1^ Constructive scientific dialogue is essential when proposing alternative therapies for pelvic venous disorders, an area in which evidence remains limited and treatment paradigms continue to evolve.

We fully agree that application of thermal energy within the retroperitoneal venous system requires meticulous technique and careful patient selection. For this reason, we designed a prospective descriptive study intended to demonstrate technical feasibility and safety rather than establish superiority over the established embolization techniques. As noted in our article, larger multicenter studies with longer follow-up and standardized energy-delivery protocols are necessary before broad adoption can be recommended.

The concern regarding thermal injury is important. Throughout all procedures, we used continuous fluoroscopic and ultrasound guidance to assess the anatomical relationship between the gonadal vein and adjacent structures (ureter and iliac vessels). We selected the 1470-nm radial laser specifically for its preferential absorption by water and reduced penetration depth compared with earlier laser technologies, which, together with a controlled pullback of 3 to 5 sec/cm, theoretically minimizes unintended tissue injury. Importantly, in our initial cohort, no ureteral, bowel, neurologic, or vascular complications were observed during the perioperative period or follow-up.

We also acknowledge the discussion regarding the absence of tumescent anesthesia. Traditional endovenous thermal ablation in superficial truncal veins relies on tumescence for vein compression, heat dissipation, and separation from adjacent tissues. However, the anatomical environment of pelvic veins differs substantially from that of the saphenous compartment. Our objective was not to replicate superficial venous ablation techniques identically, but rather to adapt thermal technology to a distinct venous territory with different hemodynamic and anatomical characteristics. Although residual intraluminal blood may partially contribute to thrombotic occlusion, this mechanism is not necessarily synonymous with procedural failure. In fact, durable fibrosis following controlled endoluminal thrombosis has been described in multiple venous interventions.

Regarding the possibility of future recanalization, we agree that long-term durability remains to be determined. Nevertheless, our early imaging follow-up demonstrated persistent occlusion and symptomatic improvement. These findings should be interpreted with caution but support continued investigation rather than abandonment of the concept. Importantly, our intention is not to promote indiscriminate use of this technique but to encourage systematic scientific evaluation in experienced venous centers.

We strongly concur that informed consent is essential whenever innovative techniques are performed. In our practice, all patients were extensively counseled regarding the investigational nature of the procedure, alternative treatment options, and the limited long-term evidence available. Institutional ethical oversight and rigorous postoperative surveillance were maintained throughout the study.

Finally, we appreciate Professor Whiteley's emphasis on scientific rigor and patient safety principles that we fully share. Innovation in vascular surgery has historically progressed through cautious, iterative development, transparent reporting, and open academic dialogue. We hope that continued collaborative investigation will clarify the role, limitations, and optimal technical parameters of endovenous thermal ablation in selected patients with pelvic venous disorders.

## Funding

None.

## Disclosures

None.

## References

[1] Moreno L., Rivero F., Forero Ramirez N., Cabrera Vargas L.F. (2026). A novel endovascular thermal ablation technique for pelvic venous disorders via basilic vein access: a prospective descriptive study. J Vasc Surg Venous Lymphat Disord.

